# Supplementary material for: Autonomous model protocell division driven by molecular replication
Source: Nat Commun. 2017 Aug 10;8:237. doi: 10.1038/s41467-017-00177-4 (PMC5552811; doi:10.1038/s41467-017-00177-4)
Supplement: Supplementary file 1 — Supplementary Information [file 41467_2017_177_MOESM1_ESM.pdf]

File Name: Supplementary Information
Description: Supplementary Figures, Supplementary Tables and Supplementary Methods

File Name: Supplementary Movie 1
Description: Video of droplet placement from reaction mixture at different reaction times using the droplet robot.

File Name: Peer Review File
Description:

Supplementary Methods

General Info

All chemicals were purchased from Sigma Aldrich, Fluorochem, TCI organics or Cambridge Isotope Laboratories and used without further purification. All NMR spectra were recorded at 25 °C. Flash chromatography was carried out on a Reveleris X2 flash chromatography system. All NMR spectra were recorded at 25 °C. NMR spectra were measured on a Bruker Avance II 400 MHz, Avance III 400 MHz, Avance III 500 MHz, or Avance III 600 MHz spectrometer. Chemical shifts are reported in ppm relative to the residual solvent peak. Chemical shifts (δ) are given in ppm and coupling constants (J) are quoted in hertz (Hz). Resonances are described as s (singlet), d (doublet), t (triplet), q (quartet), and m (multiplet). Electron impact (EI) mass spectrometry was carried out on a Jeol MStation JMS-700 High Resolution Mass Spectrometer. Electrospray ionisation (ESI) mass spectrometry was carried out on a Bruker microTOFq High Resolution Mass Spectrometer (LTQ Orbitrap XL for compounds **7** and **8**). Elemental analysis was measured with an Exeter CE-440 Elemental Analyser.

Synthesis of 4-nitro(6-bromopyridin-2-yl)benzamide

To a solution of p-nitrobenzoyl chloride (2.15 g, 11.6 mmol) in dry dichloromethane (20 mL), a solution of 2-amino-6-bromopyridine (2.0 g, 11.6 mmol) and triethylamine (1.62 mL, 11.6 mmol) in dry dichloromethane (10 mL) was added dropwise with stirring. The mixture was stirred for 18 h at room temperature. Water (20 mL) was then added. The organic layer was washed with water followed by saturated $NaHCO_3$ solution (3 x 20 mL each), dried (Na_2SO_4) and the solvent removed *in vacuo.* The crude product was recrystallized from MeOH to afford the title compound as a pale yellow crystalline solid (mp 186.9-190.5 °C) (2.48 g, 66%) δ_H (500 MHz, $CDCl_3$) 8.61 (1H, s, C(O)NH), 8.37 (2H, dt, $^3J_{HH}$ = 8.9 Hz, $^4J_{HH}$ = 2.0 Hz, ArH), 8.34 (1H, d, $^3J_{HH}$ = ArH), 8.08 (2H, dt, $^3J_{HH}$ = 8.9 Hz, $^4J_{HH}$ = 2.0 Hz, ArH), 7.66 (1H, t, $^3J_{HH}$ = 8.0 Hz, ArH), 7.31 (1H, dd, $^3J_{HH}$ = 7.7 Hz, $^4J_{HH}$ = 0.6 Hz) , δ_C (125.8 MHz, $CDCl_3$) 195.0 (s, ArCH), 176.4 (s, C(O)NH), 175.5 (s, ArC), 144.5 (s, ArC), 139.2 (s, ArC) , 137.3 (s, ArC), 128.8 (s, ArCH), 128.1 (s, ArCH), 105.4 (s, ArCH), 61.2 (s, ArC), 57.1, 54.2 (s, ArCH) MS (EI+) m/z (%) 376.321 (15) [M+], 292 (25) [Amine], 150.1 (76) [$^+C(O)PhNO_2$], 120.1 (26), [$^+C(O)PhNH_2$], 104.1 (47), 84.0 (100), 49.0 (41) HRMS: found 320.9747 (M+), $C_{12}H_8O_3N_3Br$ requires 320.9749.

Synthesis of 4-nitro(6-dodec-1-yn-yl)pyridin-2-yl-benzamide

$Pd(PPh_3)_2Cl_2$ (0.088 g, 0.13 mmol) and CuI (0.041 g, 0.21 mmol) were added to a solution of 4-nitro-(6-bromopyridin-2-yl) benzamide (1.00 g, 3.10 mmol), 1-octyne (0.90 ml, 4.20 mmol) and triethylamine (1.17 ml, 8.4 mmol) in dry THF (20 mL). The mixture was heated to reflux for 3 h under nitrogen. The mixture was then extracted with dichloromethane (20 mL), washed with 1 M KI, 0.1 M EDTA and water (3 x 20 mL each), dried ($MgSO_4$) and the solvent evaporated *in vacuo*. The crude product was applied to a 3 cm pad of silica, washed with petrol and eluted with ethyl acetate. Flash chromatography on a 40 g silica cartridge (5-20% gradient ethyl acetate/petrol over 12 column volumes) afforded the title compound as a pale yellow solid (mp 76.3-78.3 °C) (0.85 g, 67 %) δ_H (500 MHz, $CDCl_3$) 8.72 (1H, s, C(O)NH), 8.35 (2H, dt, $^3J_{HH}$ = 8.8 Hz,

$^4J_{HH}$ = 2.0 Hz, ArH), 8.29 (1H, d, $^3J_{HH}$ = 8.2 Hz, ArH), 8.07 (2H, dt, $^3J_{HH}$ = 8.9 Hz, $^4J_{HH}$ = 2.0 Hz, ArH), 7.73 (1H, t, $^3J_{HH}$ = 8.1 Hz, ArH), 7.21 (1H, d, $^3J_{HH}$ = 7.6 Hz, ArH), 2.43 (2H, t, $^3J_{HH}$ = 7.2 Hz, C≡CH_2-), 1.62 (2H, quint, $^3J_{HH}$ = 7.7 Hz, CH_2), 1.47-1.39 (2H, m, CH_2), 1.36-1.16 (13H, m, CH_2), 0.87 (3H, t, $^3J_{HH}$ = 7.1 Hz, -CH_3), δ_C (125.8 MHz, $CDCl_3$) 163.8 (s, ArC), 150.8 (s, ArC), 150.2 (ArC), 142.3 (s, ArC), 139.7 (s, ArC), 139.0 (s, ArCH), 128.5 (s, ArCH), 124.2 (s, ArCH), 123.9 (s, ArCH), 113.3 (s, ArCH), 92.2 (s, C≡C) , 92.2 (s, C≡C), 79.6 (s, -C≡C-), 32.1-19.4 (9 peaks, all CH_2), 14.3 (s, CH_3)

Synthesis of 4-amino(6-dodecyl)pyridin-2-yl-benzamide (**1**)

4-nitro(6-dodec-1-yn-yl)pyridin-2-yl-benzamide (0.813 g, 2.0 mmol) and 10% palladium on carbon (0.162 g) were suspended in ethanol (20 mL) and stirred under positive pressure of hydrogen at r.t for 36 h. The mixture was filtered through a pad of celite and the solvent removed *in vacuo*. The residue was purified by flash chromatography on a 40 g silica cartridge (0-10% gradient ethyl acetate/petrol over 8 column volumes) to afford the title compound as a white solid (mp 92.5-94.7 °C) (0.589 g, 77%). δ_H (500 MHz, $CDCl_3$) 8.34 (1H, s, C(=O)NH), 8.16 (1H, d, $^3J_{HH}$ = 8.1 Hz, ArH), 7.77 (2H, dt, $^3J_{HH}$ = 8.8 Hz, $^4J_{HH}$ = 2.2 Hz, ArH), 7.62 (1H, t, $^3J_{HH}$ = 7.7 Hz, ArH), 6.88 (1H, d $^3J_{HH}$ = 7.5 Hz, ArH), 6.71 (2H, dt, $^3J_{HH}$ = 8.8 Hz, $^4J_{HH}$ = 2.0 Hz, ArH), 4.04 (2H, s, NH_2), 2.68 (2H, t, $^3J_{HH}$ = 8.1 Hz, CH_2), 1.69 (2H, quint, $^3J_{HH}$ = 8.0 Hz, CH_2}), 1.40-1.18 (18H, m, CH_2), 0.88 (3H, t, $^3J_{HH}$ = 7.2 Hz, CH_2), δ_C (100.6 MHz, $CDCl_3$) 165.4 (s, C=O) ,160.9 (s, ArC), 151.3 (s, ArC), 150.4 (s, ArC), 138.9 (s, ArCH), 129.4 (s, ArCH), 123.7 (s, ArC), 118.4 (s, ArCH), 114.3 (s, ArCH), 111.3 (s, ArCH), 38.0(s, CH_2), 32.1 (s, CH_2), 30.0-29.0 (multiple peaks, all CH_2), 22.8 (s, CH_2), 14.3 (s, CH_3), HRMS: found 382.2835 (M+H), $C_{24}H_{36}N_3O$ requires 382.2853, Anal. Calcd (%): C: 75.55, H: 9.25, N: 11.01, Found: C: 75.47, H: 9.28, N: 10.96

Synthesis of 4-(formylphenyl)acetic acid (**2**)

2-(4-bromomethyl)phenyl)acetic acid (1.0 g, 4.4 mmol) and hexamethylenetetramine (1.84 g, 13.1 mmol)were dissolved in 50% aqueous ethanol (10 mL) and heated under reflux for 4 hours. While still under reflux, concentrated hydrochloric acid (2 mL) was added to the solution. After 15 minutes, the reaction mixture was left to cool to room temperature, diluted with water (15 mL) and extracted with dichloromethane (3 x 40 mL). The organic layers were combined, dried ($MgSO_4$) and the solvent removed *in vacuo*. The crude residue was crystallised from dichloromethane by addition of hexane to afford the pure product (463 mg, 63%) as white crystals (mp 125-129 °C, lit. 125-127 °C). δ_H (400 MHz, $CDCl_3$) 10.04 (1H, s, CHO), 7.90 (2H, dt, $^3J_{HH}$ = 8.3 Hz, $^4J_{HH}$ = 2.0 Hz, ArH), 7.50 (2H, d, $^3J_{HH}$ = 8.3 Hz, ArH), 3.77 (2H, s, $ArCH_2$) δ_C (100.6 MHz, $CDCl_3$) 192.2 (s, CHO), 176.1 (s, COOH), 140.2 (s, ArC), 135.7(s, ArC), 130.1 (s, ArCH), 130.2 (s, ArCH), 41.3 (CH_2) (EI+) m/z (%) = 164.05 (75) [M+], 119 (36) [M-CO_2+], 107 (5), 91 (100) [$C_7H_7^+$], 65 (16) HRMS: found 164.0471 [M^+], $C_9H_8O_3$ requires 164.0473.

Synthesis of amphiphilic imine template (**3**)

4-Amino-N-(6-dodecylpyridin-2-yl)benzamide (115 mg, 0.3 mmol) and 4-formyl phenylacetic acid (49.2 mg, 0.3 mmol) were suspended in dry ethanol (3.0 mL) and heated to 100 °C in a sealed tube by microwave for 2 h. The resulting solid was collected

by filtration and washed with ice-cold ethanol to afford the title compound (53 mg, 34 %) as a pale yellow solid (mp 159.2 -160.6 °C) δ_H (400 MHz, $CDCl_3$) 10.97 (1H, br s, CO_2H), 8.43 (1H, s, CH), 8.31 (1H, d, $^3J_{HH}$ = 8.3 Hz), 7.95 (2H, d, $^3J_{HH}$ = 8.6 Hz, ArH), 7.93 (2H, d, $^3J_{HH}$ = 8.2 Hz, ArH), 7.77 (1H, t, $^3J_{HH}$ = 8.0 Hz, ArH), 7.41 (2H, d, $^3J_{HH}$ = 8.2 Hz, ArH), 7.04 (2H, d, $^3J_{HH}$ = 8.6 Hz, ArH), 6.98 (1H, d, $^3J_{HH}$ = 7.5 Hz, ArH), 3.81 (2H, s, CH_2), 2.76 (2H, t, $^3J_{HH}$ = 7.7 Hz, CH_2), 1.70 (2H, quint, $^3J_{HH}$ = 7.5 Hz, CH_2), 1.44-1.20 (18H, m, CH_2), 0.88 (3H, t, $^3J_{HH}$ = 7.0Hz, CH_3), δ_C (150.9 MHz, $CDCl_3$) 177.0 (s, CO_2H), 166.7 (s, C(O)NH), 160.9 (s, ArC), 160.4 (s, ArC), 155.3 (s, ArC), 151.7 (s, ArC), 140.4 (s, ArCH), 138.5 (s, ArCH), 134.8 (s, ArC), 131.3 (s, ArCH), 130.5 (s, ArC), 129.5 (s, ArCH), 129.1 (s, ArCH), 120.8 (s, ArCH), 119.1 (s, ArCH), 113.8 (s, CH=N), 40.7 (s, CH_2), 36.2 (s, CH_2), 32.1 (s, CH_2), 32.0 (s, CH_2), 30.4, multiple peaks between 30.0 and 29.0 (all CH_2), 22.8 (s, CH_2), 14.3 (s, CH_3), HRMS: found 550.3014 (M+Na), $C_{33}H_{42}N_3O_3$ requires 550.3007, Anal. Calcd (%): C: 75.11, H: 7.83, N: 7.96, Found: C: 75.20, H: 7.89, N: 8.02

Synthesis of 3-pentyl-1-undecyne

To a solution of 1-octyne (4.40 g, 0.04 mmol) in dry hexane (40 mL) at -78 °C, n-butyllithium (2.5 M in hexanes, 40 mL) was added dropwise with rapid stirring. After stirring for 30 min, the solution was warmed to – 47 °C in a dry ice/acetonitrile bath, and 1-bromooctane (7.32 g, 0.04 mol) was then added dropwise. The solution was allowed to warm to room temperature over 24 h. HCl (6M, 60 mL) was slowly added to the mixture in an ice bath. The organic phase was separated and washed with water, dried ($MgSO_4$) and the solvent removed *in vacuo*. The residue was dissolved in ethanol (20 mL) in a microwave reactor tube and pyridine (1.1 equiv) was added. The mixture was heated to 120 °C by microwave for 2.5 h. The resulting mixture was filtered and the volatiles removed *in vacuo*. The residue was purified on a 5 cm silica plug, eluting with hexane to afford the title compound (3.90 g, 44%) as a pale yellow oil which was carried forward to the next step. δ_H (400 MHz, $CDCl_3$) 2.30 (1H, m, C≡C-CH), 2.01 (1H, d, $^4J_{HH}$ = 2.40, C≡CH), 1.60-1.10 (22H, m, CH_2), 0.94-0.84 (6H, m, CH_3)

Synthesis of 4-Nitro(6-(3-pentyl-1-undec-yn-yl)pyridin-2-yl-benzamide

$Pd(PPh_3)_2Cl_2$ (0.131 g, 0.186 mmol) and CuI (0.029 g, 0.154 mmol) were added to a solution of -4-nitro-(6-bromopyridin-2-yl) benzamide (1.00 g, 3.1 mmol), 3-pentyl-1-undecyne (0.93 g, 4.19 mmol) and triethylamine (0.89 ml, 12.4 mmol) in dry THF (30 mL). The mixture was heated to reflux for 3 h under nitrogen, then extracted with dichloromethane, washed with 1 M KI solution, 0.1 M EDTA solution and brine (3 x 30 mL each), dried ($MgSO_4$) and the volatiles removed *in vacuo*. The resulting oil was applied to a 5 cm silica pad, washed with petrol and eluted with ethyl acetate. The residue was purified by flash chromatography on a 40 g silica cartridge (0-10% ethyl acetate/petrol over 10 column volumes) to afford the title compound (0.735 g, 51 %) as a pale yellow oil. δ_H (500 MHz, $CDCl_3$) 8.79 (1H, br s, C(=O)NH), 8.34 (2H, dd, $^3J_{HH}$ = 8.5 Hz, $^4J_{HH}$ = 1.6 Hz, ArH), 8.28 (1H, d, $^3J_{HH}$ = 8.5 Hz, ArH), 8.08 (2H, dt, $^3J_{HH}$ = 8.5 Hz, $^4J_{HH}$ = 2.4 Hz, ArH), 7.73 (1H, t, $^3J_{HH}$ = 8.0 Hz, ArH), 7.22 (1H, d, $^3J_{HH}$ = 7.3 Hz), 2.55 (1H, m, ≡C-CH), 1.60-1.10 (22H, m, CH_2), 0.89 (3H, t, $^3J_{HH}$ = 6.7 Hz, CH_3), 0.87 (3H, t, $^3J_{HH}$ = 6.7 Hz, CH_3), δ_C (125 MHz, $CDCl_3$) 163.7 (s, C=O), 150.8 (s, ArC-NO_2),

150.0 (s, ArC), 142.2 (s, ArC), 139.6 (s, ArC), 138.8 (s, ArCH), 128.5 (s, ArCH), 124.0 (s, ArCH), 113.4 (s, ArCH), 95.7 (s, ArCH), 80.5 (s, ArC-C≡), 34.8 (s, ≡C), 34.7 (s, ≡CH), 32.3 (s, CH) 31.9 (s, CH_2), 31.7 (s, CH_2), 30.1 (s, CH_2), 29.5 (s, CH_2), 29.3 (s, CH_2), 27.5 (s, CH_2), 27.1 (s, CH_2), 22.7 (s, CH_2), 22.7 (s, CH_2), 14.1 (s, CH_3), 14.1 (s, CH_3), HRMS: found 486.2727 (M+Na), $C_{28}H_{37}N_3O_3Na$ requires 486.2707

Synthesis of 4-amino(6-(3-pentyl-1-undec-yn-yl)pyridin-2-yl-benzamide (**7**)

4-Nitro(6-(3-pentyl-1-undec-yn-yl)pyridin-2-yl-benzamide (0.652 g, 1.41 mmol) and 10% palladium on carbon (0.13 g) were suspended in ethanol (15 mL) and stirred under positive pressure of hydrogen for 24 h. The mixture was filtered through a pad of celite and the volatiles removed *in vacuo*. The residue was purified by flash chromatography on a 12 g silica cartridge (0-20% ethyl acetate/petrol over 18 column volumes) to afford the title compound (0.493 g, 81%) as a pale yellow solid (mp 48.7-50.8 °C). δ_H (500 MHz, $CDCl_3$) 8.49 (1H, s, C(=O)NH), 8.29 (1H, d, $^3J_{HH}$ = 8.5 Hz, ArH), 7.73 (2H, dt, $^3J_{HH}$ = 8.7 Hz, $^4J_{HH}$ = 1.9 Hz, ArH), 7.65 (1H, t, $^3J_{HH}$ = 7.5 Hz, ArH), 7.13 (1H, d, $^3J_{HH}$ = 7.5 Hz, ArH), 6.69 (2H, dt, $^3J_{HH}$ = 8.6 Hz, $^4J_{HH}$ = 1.7 Hz, ArH), 4.10 (2H, s, NH_2), 2.60-2.51 (1H, m, CH), 1.60-1.18 (22H, m, CH_2), 0.89 (3H, t, $^3J_{HH}$ = 7.0 Hz, CH_3), 0.87 (3H, t, $^3J_{HH}$ = 7.2 Hz, CH_3) δ_C (125 MHz, $CDCl_3$) 165.4 (s, C=O), 151.8 (s, ArC-NH_2), 150.5 (s, ArC), 142.0 (s, ArC), 138.5 (s, ArC), 129.5 (s, ArCH), 123.4 (s, ArCH), 123.0 (s, ArCH), 114.3 (s, ArCH), 113.1 (s, ArCH), 94.9 (s, C≡C), 81.0 (s, C≡C), 35.0 (s, CH_2), 34.9 (s, CH_2), 32.4 (s, CH_2), 32.0 (s, CH_2), 31.9 (s, CH_2), 31.1 (s, CH_2), 29.7 (s, CH_2), 29.6 (s, CH_2), 29.5 (s, CH_2), 27.6 (s, CH_2), 27.3 (s, CH_2), 22.8 (s, CH_2), 22.7 (s, CH_2), 14.24 (s, CH_3), 14.23 (s, CH_3), HRMS: found 456.2978 (M+Na) $C_{28}H_{37}N_3ONa$ requires 456.2985, Anal. Calcd. (%): C: 77.54, H: 9.07, N: 9.69, Found: C: 77.60, H: 9.15, N: 9.66.

Synthesis of Branched template imine (**8**)

4-amino(6-(3-pentyl-1-undec-yn-yl)pyridin-2-yl-benzamide (0.131 g, 0.3 mmol) and 4-formyl phenylacetic acid (0.049 g, 0.3 mmol) were suspended in dry ethanol (3.0 mL) with a few 4 Å molecular sieve beads. The solution was heated to 100 °C in a sealed tube by microwave for 2 h. The resulting solid was collected by filtration and washed with ice cold ethanol to afford the title compound as a pale yellow solid (mp 160.6-162.7 °C) (0.117 g, 67%) δ_H (400 MHz, $CDCl_3$) 10.89 (1H, br s, COOH), 8.44(1H, dd, $^3J_{HH}$ = 8.9 Hz, $^4J_{HH}$ = 1.1 Hz, ArH), 8.43 (1H, s, CH=N), 7.98 (2H, dt, $^3J_{HH}$ = 8.7 Hz, $^4J_{HH}$ = 2.0 Hz, ArH), 7.94 (2H, d, $^3J_{HH}$ = 8.22, ArH), 7.75 (1H, t, $^3J_{HH}$ = 8.0 Hz, ArH), 7.42 (2H, d, $^3J_{HH}$ = 8.2 Hz, ArH), 7.18 (1H, dd, $^3J_{HH}$ = 7.5 Hz, $^4J_{HH}$ = 0.9 Hz), 7.05 (2H, dt, $^3J_{HH}$ = 8.6 Hz, $^4J_{HH}$ = 2.0 Hz, ArH), 3.81 (2H, s, CH_2), 2.60 (1H, quint, $^3J_{HH}$ = 4.8 Hz, CH), 1.72-1.18 (22H, m, CH_2), 0.94 (3H, t, $^3J_{HH}$ = 6.9 Hz, CH_3), 0.88 (3H, t, $^3J_{HH}$ = 7.3 Hz, CH_3), δ_C (125 MHz, $CDCl_3$) 166.8 (s, C(=O)N), 161.0 (s, C=N), 155.6 (s, ArC), 148.2 (s, ArC), 141.1 (ArC), 139.8 (s, ArCH), 138.3 (s, ArC), 135.1 (s, ArC), 131.4 (s, ArC), 130.7 (s, ArCH), 129.7 (s, ArCH), 129.3 (s, ArCH), 128.6 (s, ArCH), 123.3 (s, ArCH), 120.9 (s, ArCH), 100.3 (s, C≡C), 97.6 (s, C≡C), 40.5 (s, CH_2), 35.1 (s, CH_2), 32.7 (s, CH_2), 32.2 (s, CH), multiple peaks between 32.0 and 22.0 (all CH_2), 14.4 (s, CH_3), HRMS: found 578.3373 (M-H), $C_{37}H_{44}N_3O_3$ requires 578.3388, Anal. Calcd. (%): C: 76.65, H: 7.82, N: 7.25, Found: C: 76.39, H: 7.82, N: 7.30

Kinetics procedure

All solutions were made up in dry $CDCl_3$ (redried with further 4 Å molecular sieves), and kept in a dessicator. A screw-cap NMR tube was used to minimise evaporation and exposure to atmospheric moisture. All weighings were carried out with a six decimal place microbalance. A 20 mM solution of the amine was prepared with ~ 1 mM hexamethylbenzene as standard, and the exact concentration recorded. A 20 mM solution of 4-formyl phenylacetic acid was also prepared. For the seeded experiments, the 20 mM amine solution also contained 3.0 mM of **3**. For the reaction of **1** with p-tolualdehyde (**6**), 10 mM of 4-bromophenylacetic acid was also added to keep the acidity constant. The amine solution (0.4 mL) was transferred to a screw cap NMR tube, and spectra of the starting materials obtained. The aldehyde solution (0.4 mL) was then added, and the tube was promptly transferred to a Bruker Avance III 500 MHz NMR spectrometer and the first spectrum acquired. Spectra were acquired every 10 minutes, for a period of 18 h (unseeded) or 16 h (seeded), totalling 108 or 96 spectra respectively. The aromatic peaks that did not overlap, along with the imine peak, were integrated using the intser function Topspin 3.5. The hexamethylbenzene peak was used as an internal standard, integrals for each spectrum were individually calibrated to the hexamethylbenzene peak (i.e. no global standard was used). Times were extracted from the Topspin dataset list.

Automated periodic droplet experiments

Droplet experiments were carried out on an in-house built liquid handling robot equipped with 250 µl syringes, an improved version of the chemorobotic platform used in previous work by this lab(17). 800 µl aliquots of a 20 mM solution of both the amine under study and 4-formylphenylacetic acid (**2**) were mixed in a 1.7 ml vial capped with a PTFE/silicone rubber septum, to which Sudan blue II (3.0 mg) had been added. The robot would then take 30 µl into a glass syringe with a needle through the septum and place four 4.0 µl droplets (in a square pattern) into a 35 mm diameter petri dish containing 2.5 mL of dilute NaOH solution (pH 12.2). A webcam underneath the petri dish recorded video of the droplets for 120 s, then the dish was cleaned with distilled water and acetone, and blown dry with a ducted fan. The droplet placement needle was automatically rinsed in a septum-capped vial containing dry chloroform and 4 Å molecular sieves. This was repeated every 10 minutes for 30 experiments. From each video, the final frame was taken and the number and total area of droplets was determined using an ImageJ macro. The number of pixels was converted to mm^2 by dividing the known area of the dish in millimetres by the observed area of the dish from the image in pixels, and multiplying this value by the droplet area in pixels.

Dynamic Light Scattering

Dynamic light scattering (DLS) measurements were performed using a Brookhaven Instrument spectrometer with solid state laser (λ=532 nm) and BI-900AT multichannel digital correlator. Results were later analysed using CONTIN method.

For investigating the roles of amphiphilic imine **3** and unmodified imine **5** in encapsulating the trace amount of water in chloroform within the reverse micelles (**Supplementary Figure 6**), 2.0 mM solutions of each compound were prepared in a glove box while equal amounts of DI water at pH12.2 (0.5% v/v) were introduced into

the solution. The two vials were sealed and mixed thoroughly for a few minutes and then sonicated for 1 minute before measurement with time resolved DLS.

To follow the change in scattering intensity over the course of the self-replication reaction, both compounds were dissolved in anyhdrous chloroform separately followed by vortexing, sonication and 30 minutes of rapid stirring, then both solutions were mixed in a sealed vial, filtered with a 0.2 µm hydrophobic filter (Millipore-Millex-FG) and sealed properly in a glove box. The reaction took place in a dark environment at room temperature with moderate stirring for 36 h. The sealed reaction vial was then opened, and aliquots of water (pH12.3) up to 1.25, 2.5 and 5 µl were added to 1 ml of the solution, followed by 1 minute of sonication. The scattered intensity was then measured **(Supplementary Table 2).**

Kinetic simulation and fitting

Kinetic simulation and fitting was carried out using Berkeley Madonna. The model used was the minimal replicator model, adapting the SimFit equations from fitting of a similar experiment(12) to our software. An example command file is included. The variable parameters were the forward rate constant for the bimolecular reaction (**k_1f**), the forward and reverse rate constants for the reaction of the ternary complex (**k_6f** and **k_6r**), the association constant of the template dimer (**K_{TT}**), and the binding constant between amidopyridine and carboxylic acid sites (**$K_{complex}$**). This makes the assumption that there is no significant cooperativity in the binding of the substrates to the template. The best fit parameter values for each dataset are recorded in **Supplementary Table 1.**

The value of **k_1f** for the unseeded reaction and the unmodified control are similar to the literature value for this system (4.06 x 10^{-4} $M^{-1}s^{-1}$). For the value of **k_6f**, we obtained values for the unseeded and seeded amphiphilic replicator reactions of approximately twice the value of those from the unmodified control and from the literature (1.7 x 10^{-2} $M^{-1}s^{-1}$ vs. 8.14 x 10^{-3} $M^{-1}s^{-1}$). However, the values of **$K_{complex}$** for the amphiphilic replicator experiments were significantly lower than that for the unmodified control, which would necessitate a faster rate of reaction of the complex once substrate had bound to the template. Values of **K_{tt}** were comparable to the literature values of 65000±8000 M^{-1}.

Kinetic Model File for Berkeley Madonna

```
{1: A+B <--> T+W}
RXN1 = K1f*A*B - K1r*T*W
K1f = 1.36e-4
K1r =K1f/Kb
INIT A = 10
INIT B = 10
INIT T = 0
INIT W = 10
d/dt(A) = -RXN1-RXN2-RXN5-RXN8-RXN10+0*RXN11-RXN13
d/dt(B) = -RXN1-RXN3-RXN4-RXN8-RXN9+0*RXN12-RXN14
d/dt(T) = +RXN1-RXN2-RXN3
2*RXN7+2*RXN9+2*RXN10+RXN11+RXN12+RXN13+RXN14
d/dt(W) = +RXN1+RXN6+RXN9+RXN10+RXN11+RXN12+RXN13+RXN14
```

```
{ 2: A+T <--> AT }
RXN2 = K2f*A*T - K2r*AT
K2f =  KBf
K2r = KBr
INIT AT = 0
d/dt(AT) = +RXN2-RXN4-RXN9+RXN13

{ 3: B+T <--> BT }
RXN3 = K3f*B*T - K3r*BT
K3f =  KBf
K3r = KBr
INIT BT = 0
d/dt(BT) = +RXN3-RXN5-RXN10+RXN14

{ 4: B+AT <--> ABT }
RXN4 = K4f*B*AT - K4r*ABT
K4f =  KBf
K4r = KBr
INIT ABT = 0
d/dt(ABT) = +RXN4+RXN5-RXN6-RXN13-RXN14

{ 5: A+BT <--> ABT }
RXN5 = K5f*A*BT - K5r*ABT
K5f =  KBf
K5r = KBr

{ 6: ABT <--> TT+W }
RXN6 = K6f*ABT - K6r*TT*W
K6f = 8.14e-3
K6r = 1.82e-3
INIT TT = 0
d/dt(TT) = +RXN6+RXN7

{ 7: T+T <--> TT }
RXN7 = K7f*T*T - K7r*TT
K7f =1e3
K7r =K7f/Ktt

{ 8: A+B <--> AB }
RXN8 = K8f*A*B - K8r*AB
K8f = 1e3
K8r =K8f/KAB
INIT AB = 0
d/dt(AB) = +RXN8-RXN11-RXN12

{ 9: AT+B <--> 2T+W }
```

RXN9 = K9f*AT*B - K9r*T^2*W
K9f = Kterf
K9r = Kterr

{ 10: BT+A <--> 2T+W }
RXN10 = K10f*BT*A - K10r*T^2*W
K10f = Kterf
K10r =Kterr
{ 11: AB+A <--> T+A+W }
RXN11 = K11f*AB*A - K11r*T*A*W
K11f = Kterf
K11r =Kterr

{ 12: AB+B <--> T+B+W }
RXN12 = K12f*AB*B - K12r*T*B*W
K12f = Kterf
K12r = Kterr

{ 13: ABT+A <--> T+AT+W }
RXN13 = K13f*ABT*A - K13r*T*AT*W
K13f = Kterf
K13r =Kterr

{ 14: ABT+B <--> T+BT+W }
RXN14 = K14f*ABT*B - K14r*T*BT*W
K14f = Kterf
K14r = Kterr

{The association constant between A and B}
KAB = Kcomplex

{The equilibrium constant for the bimolecular reaction}
Kb = 0.297

{The association constant for the template dimer}
Ktt = 65000

{The association constant for template/substrate complexes}
Kcomplex = 50

KBf = 1
KBr= KBf/Kcomplex

{Bimolecular reactions through complexes}
Kterf = K1f
Kterr = Kterf/Kb

```
STARTTIME = 0
STOPTIME=63157
DT = 60

{Add up all of the template species to give final [T]}
P = T +2*TT + AT + BT + ABT

{Convert from moles to millimoles}
Pmm = P * 1000
```

ImageJ Macro for Droplet Analysis

```
setTool("oval");
\\ Top left corner coordinates and dimensions of binding box rectangle
makeOval(151, 64, 384, 382);
run("Crop");
setBackgroundColor(0, 0, 0);
run("Clear Outside");
run("Subtract Background...", "rolling=100 light");
run("HSB Stack");
setSlice(3);
run("Delete Slice");
setSlice(1);
run("Delete Slice");

setAutoThreshold("Default");
run("Threshold...");
setThreshold(95, 255);
setOption("BlackBackground", false);
run("Convert to Mask", "method=Default background=Light");

run("Fill Holes");
run("Watershed");

run("Analyze Particles...", "size=5-Infinity show=Outlines display exclude summarize slice");
currDialog = getInfo("window.type");
if(currDialog != "ResultsTable") {
close("Particle*");
```

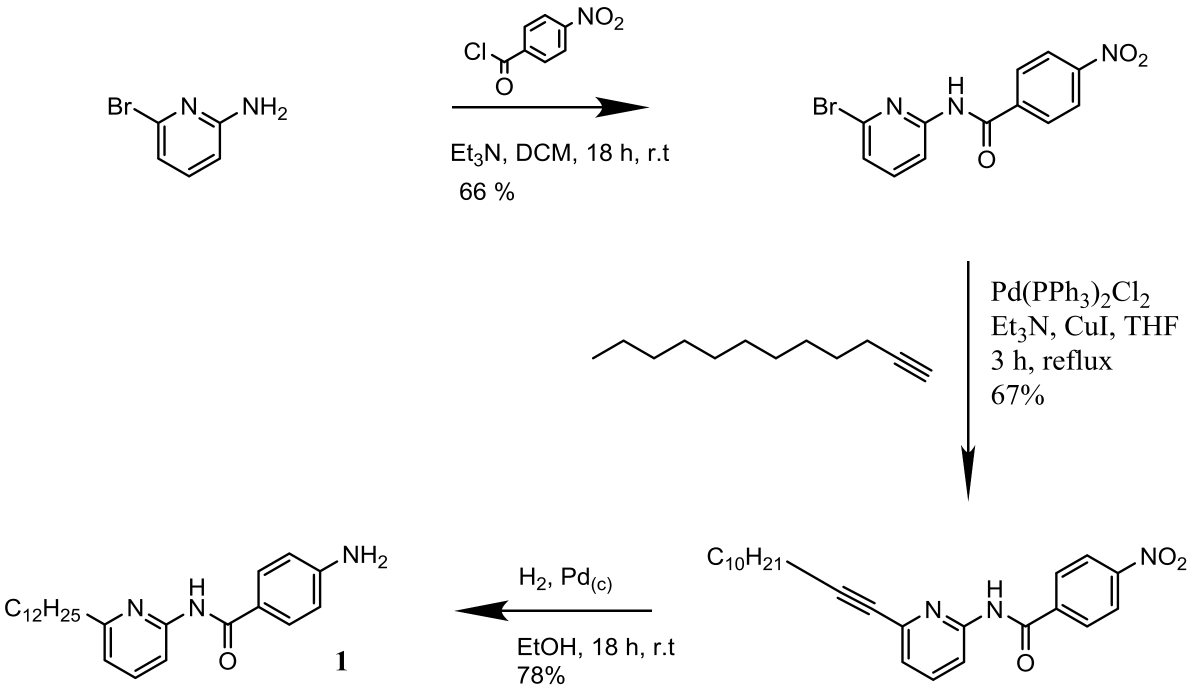

Supplementary Figure 1 - Synthesis of hydrophobic amine **1**

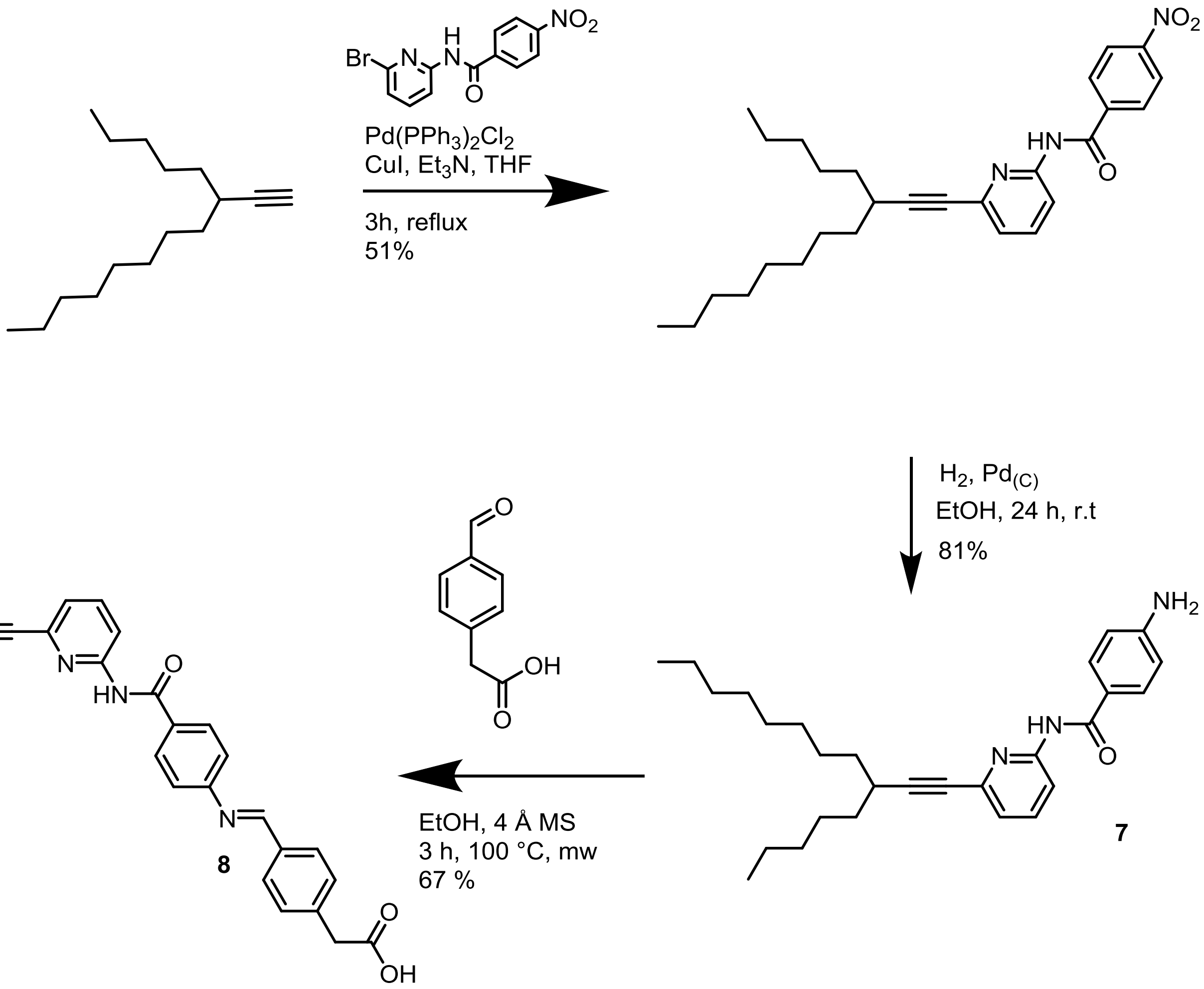

Supplementary Figure 2 - Synthesis of 3-pentyl-(undec-1-yn-)yl substituted amine **7**

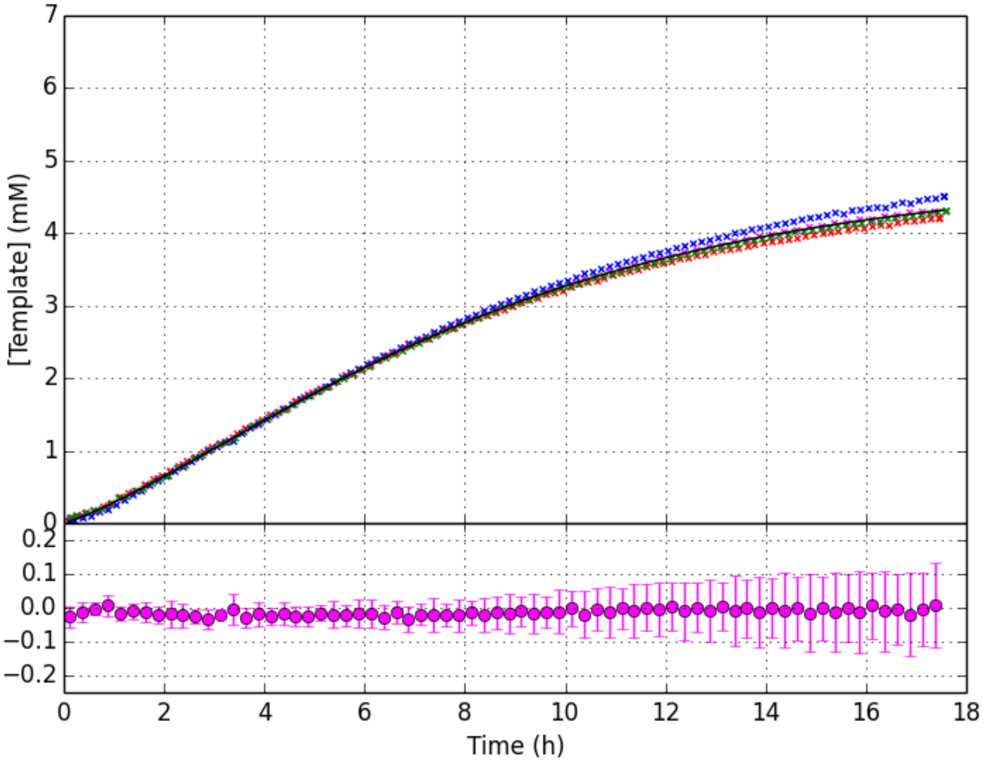

Supplementary Figure 3 - Concentration of amphiphilic imine **3** *vs* time for the reaction with no initial template added. All three individual repeats of this reaction (blue, green, red) Rolling average of all datapoints at 15 minute intervals (magenta). Simulated concentrations from best-fit model parameters (black). Residual error between the rolling average and the model fit (below, magenta). Error bars are the standard deviation of the rolling average.

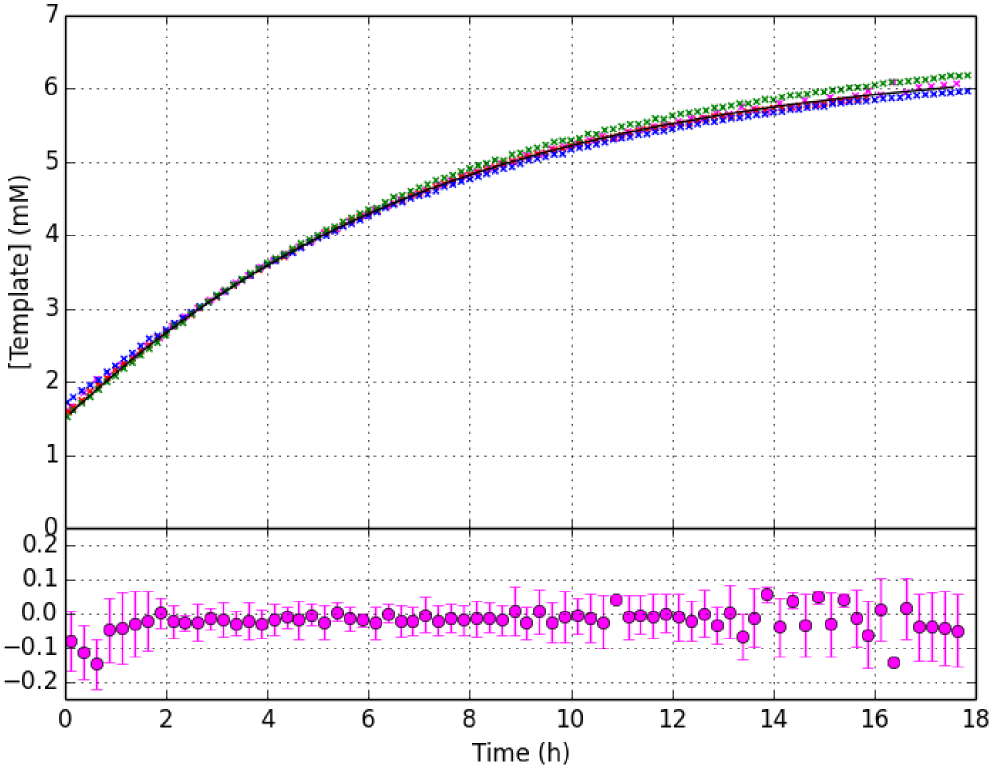

Supplementary Figure 4 - Concentration of amphiphilic imine **3** vs time for the reaction with 1.5 mM **3** added at the start. All three individual repeats of this reaction (blue, green, red). Rolling average of all datapoints at 15 minute intervals (magenta). Simulated concentrations from best-fit model parameters (black). Residual error between the rolling average and the model fit (below, magenta). Error bars are the standard deviation of the rolling average.

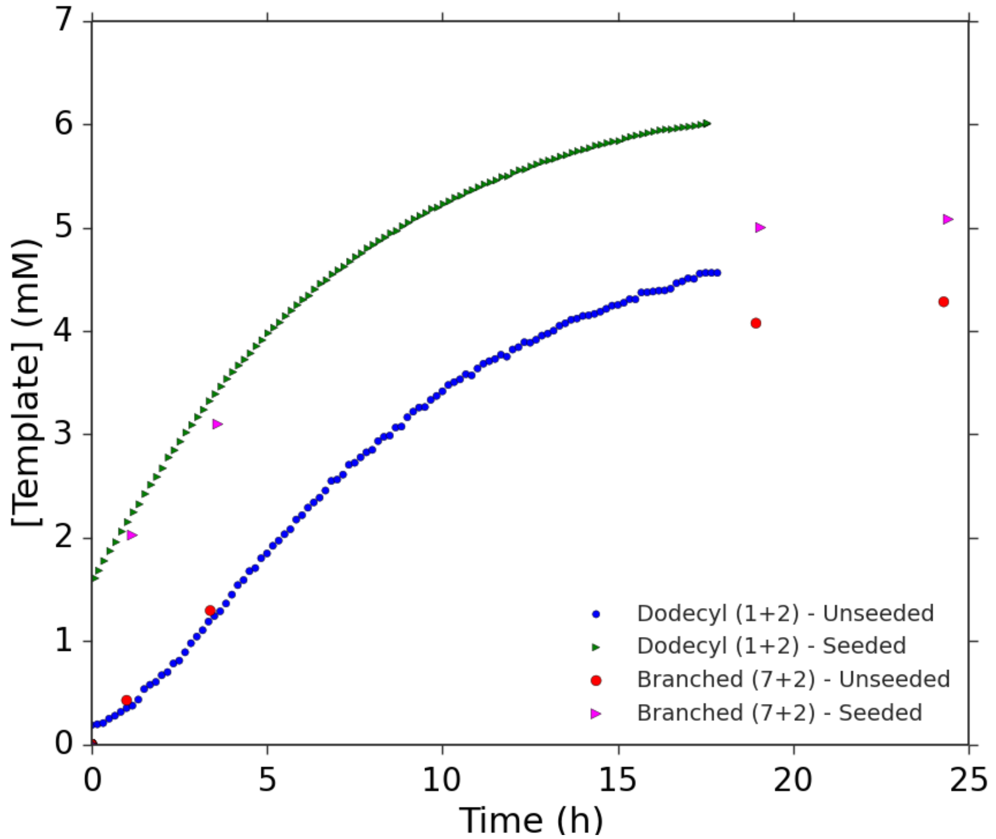

Supplementary Figure 5 - Single point experiments of reactions of 10 mM branched amine (**7**) with 10 mM 4-formyl phenylacetic acid (**2**). The unseeded reaction (red triangles) is compared to the unseeded reaction of (**1**) and (**2**) (green circles), while the reaction seeded with 1.5 mM (**8**) (blue triangles) is compared to the reaction of (**1**) and (**2**) seeded with 1.5 mM (**3**) (yellow circles).

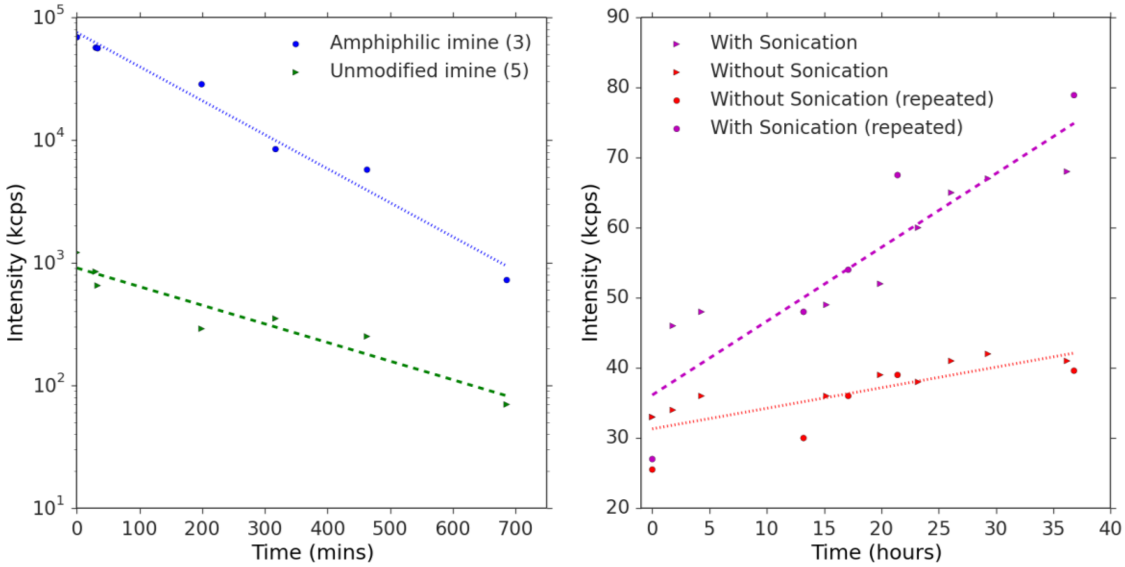

Supplementary Figure 6 - Change in scattered intensity with time for 2.0 mM solutions of amphiphilic imine **3** and unmodified imine **5** in chloroform containing 0.5 % H_2O at pH 12.2 solution, due to phase separation and imine hydrolysis. **3** was shown to be a superior surfactant to **5** for the stabilization of water in chloroform due to its initial scattering intensity being 56 times higher. b) - Change in scattered intensity over time for a mixture of **1** and **2** at 20 mM concentration in chloroform, before and after sonication. The scattering intensity of the solution increased up to around 30 hours as the reaction approached completion. The relatively low scattered intensity of the solution is a consequence of the very low percentage of water in the final state, and this was confirmed by adding more water after the completion of the reaction increasing the scattered intensity which suggests the formation of more colloidal particles in solution (**Supplementary Table 2**).

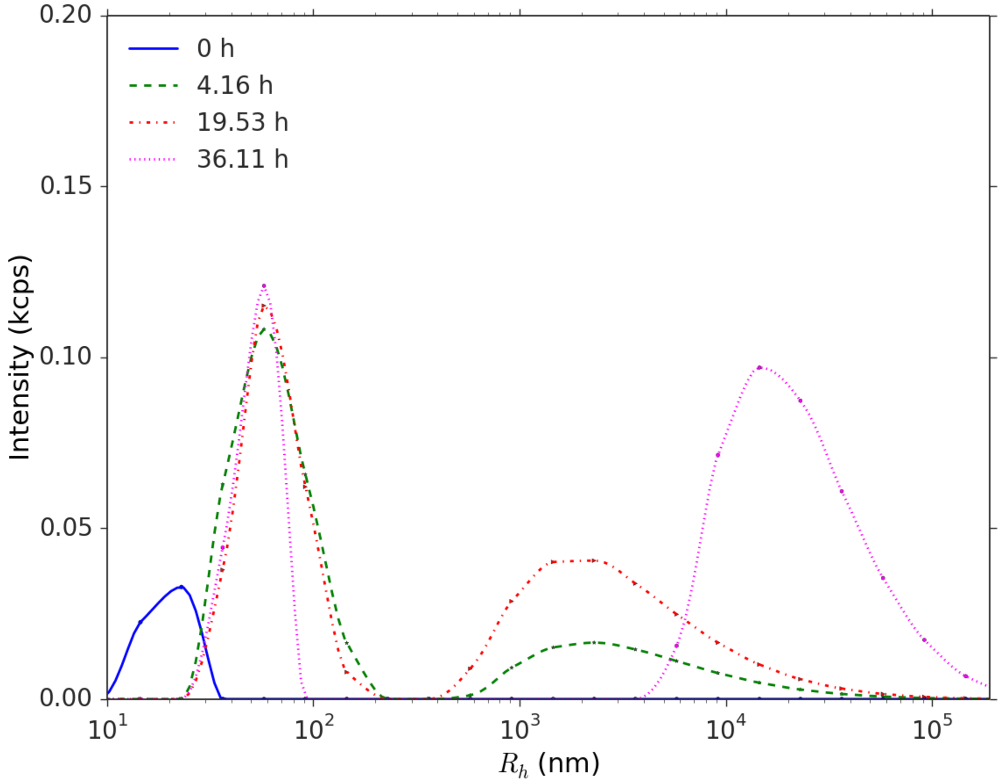

Supplementary Figure 7 - Size distribution of reverse micelles as the reaction proceeds toward completion. Sub-100 nm colloidal particles are related to reverse micelles while larger particles correspond to phase-separated traces of water.

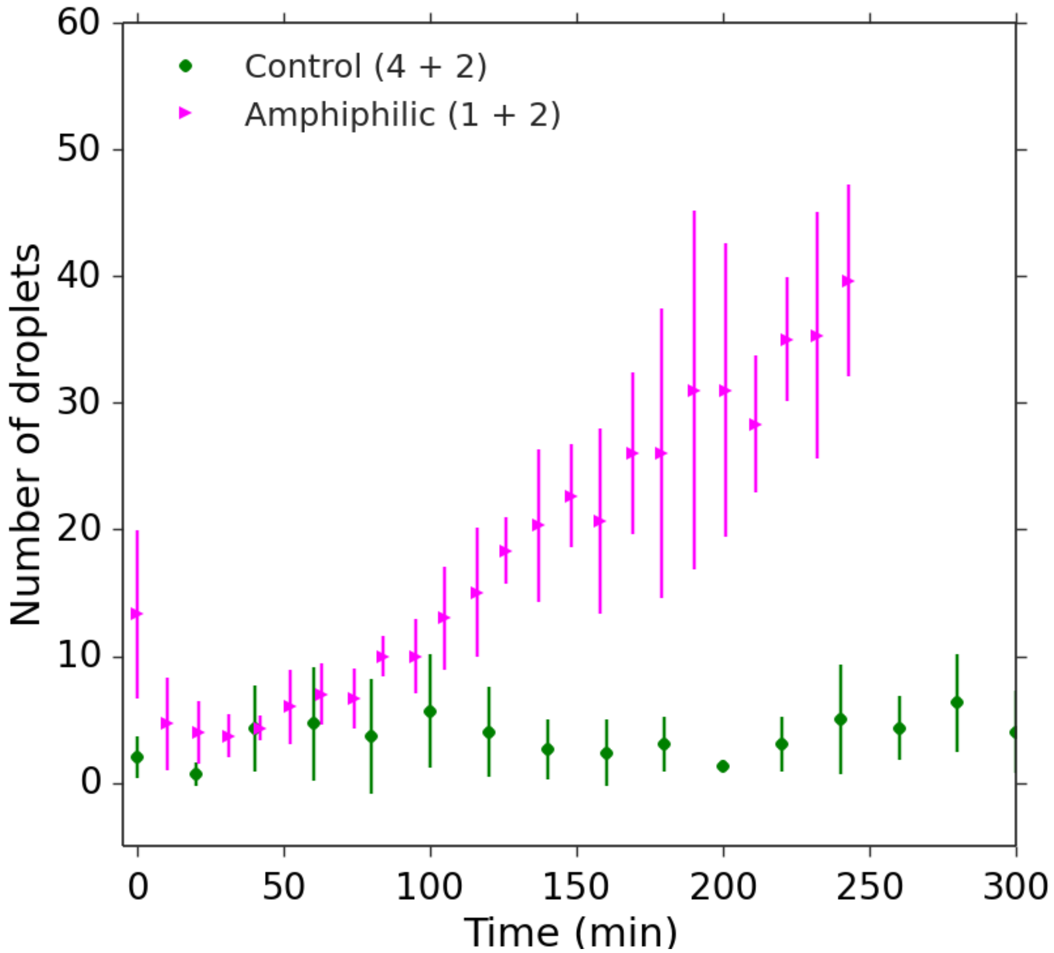

Supplementary Figure 8 – Droplet count *vs* time, for the self-replicating amphiphile forming reaction starting from 10 mM hydrophobic amine **1** with 10 mM 4-formyl(phenyl)acetic acid 2 (magenta) and for the control reaction starting from 10 mM unmodified amine 4 and 10 mM 4-formyl(phenyl)acetic acid **2**. Points are the mean value obtained from three repeats, and the error bars show the standard deviation.

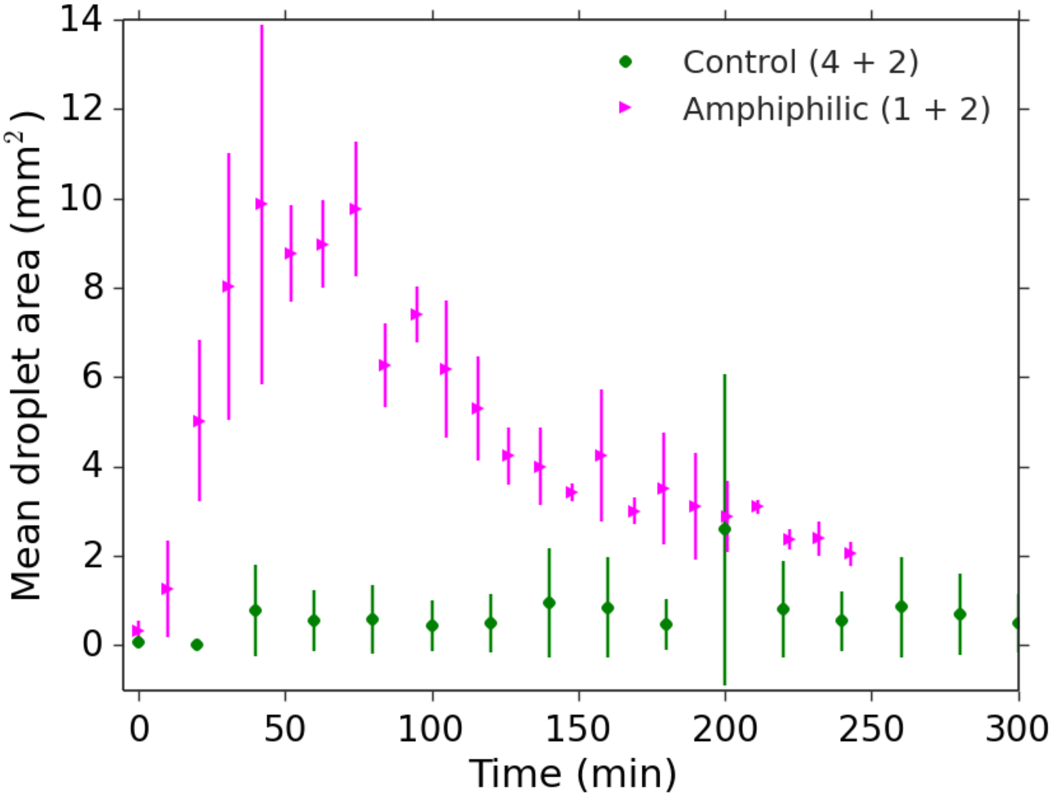

Supplementary Figure 9 – Mean droplet area *vs* time, for the self-replicating amphiphile forming reaction starting from 10 mM hydrophobic amine **1** with 10 mM 4-formyl(phenyl)acetic acid 2 (magenta) and for the control reaction starting from 10 mM unmodified amine 4 and 10 mM 4-formyl(phenyl)acetic acid **2**. Points are the mean value obtained from three repeats, and the error bars show the standard deviation.

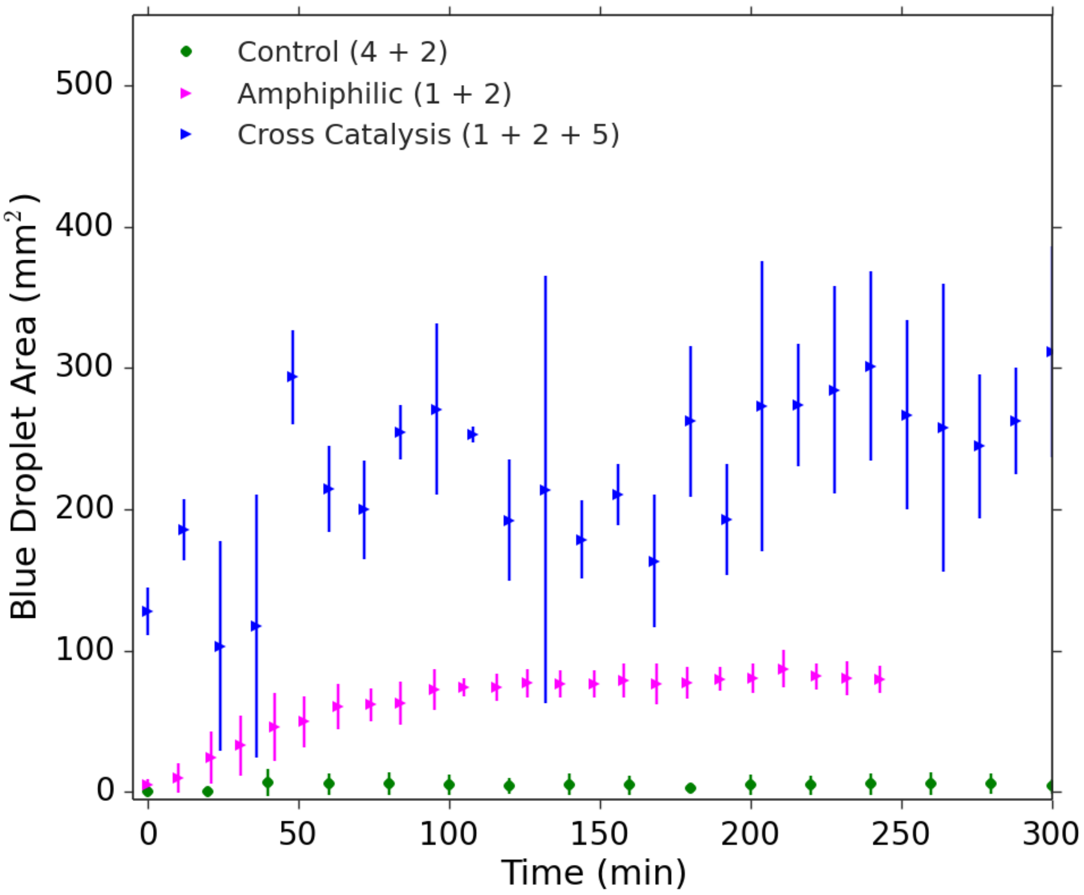

Supplementary Figure 10 - Droplet area vs time, for the cross-catalysis experiment starting from 10 mM hydrophobic amine **1**, 10 mM 4-formly phenylacetic acid **2**, and 1.5 mM unmodified replicator imine **5** (blue). Also shown is the reaction starting from 10 mM hydrophobic amine **1** with 10 mM 4-formyl(phenyl)acetic acid **2** (magenta) and the control reaction starting from 10 mM unmodified amine 4 and 10 mM 4-formyl(phenyl)acetic acid **2**. Points are the mean value obtained from three repeats, and the error bars show the standard deviation.

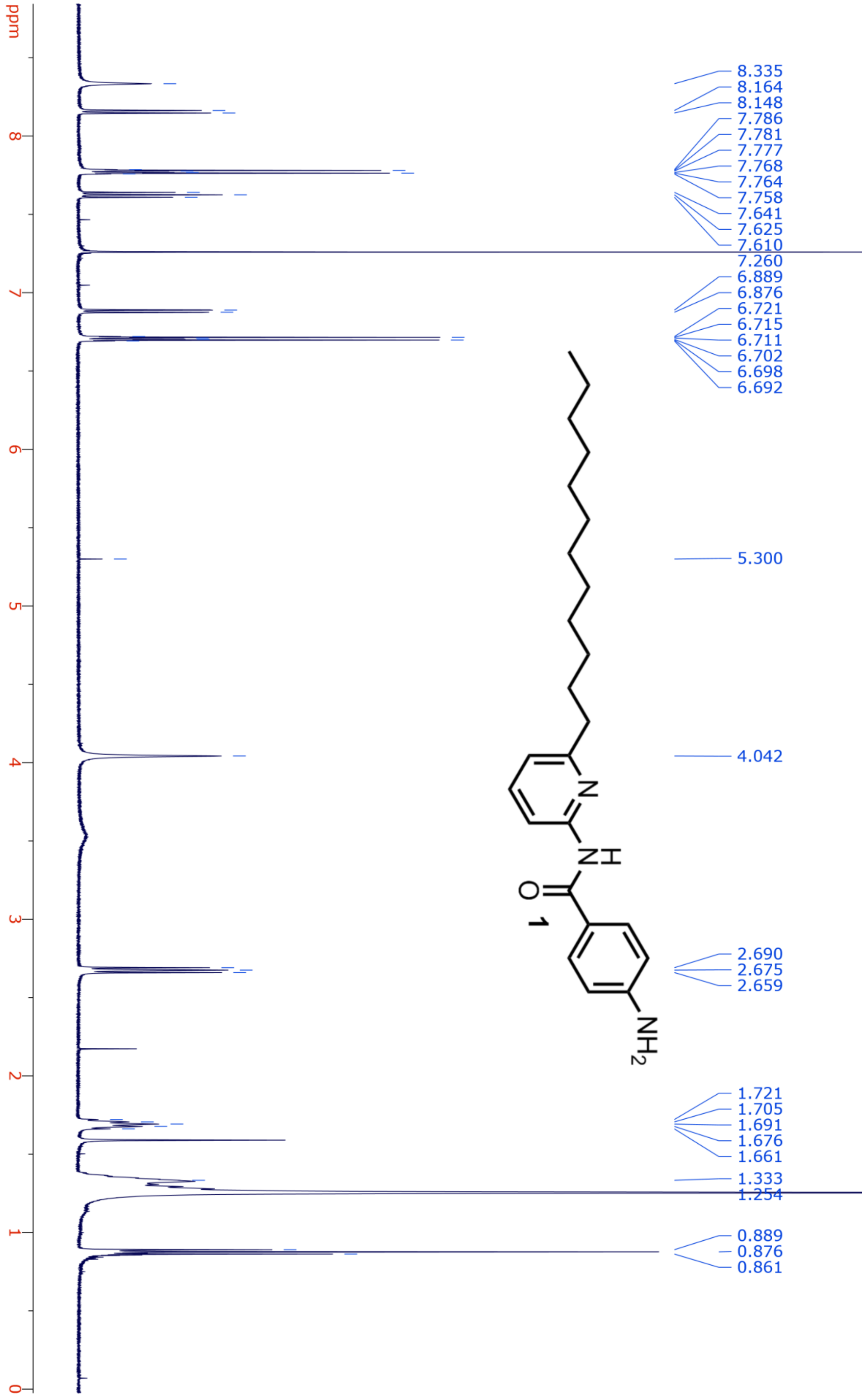

Supplementary Figure 11 – ^{1}H NMR spectrum of **1**

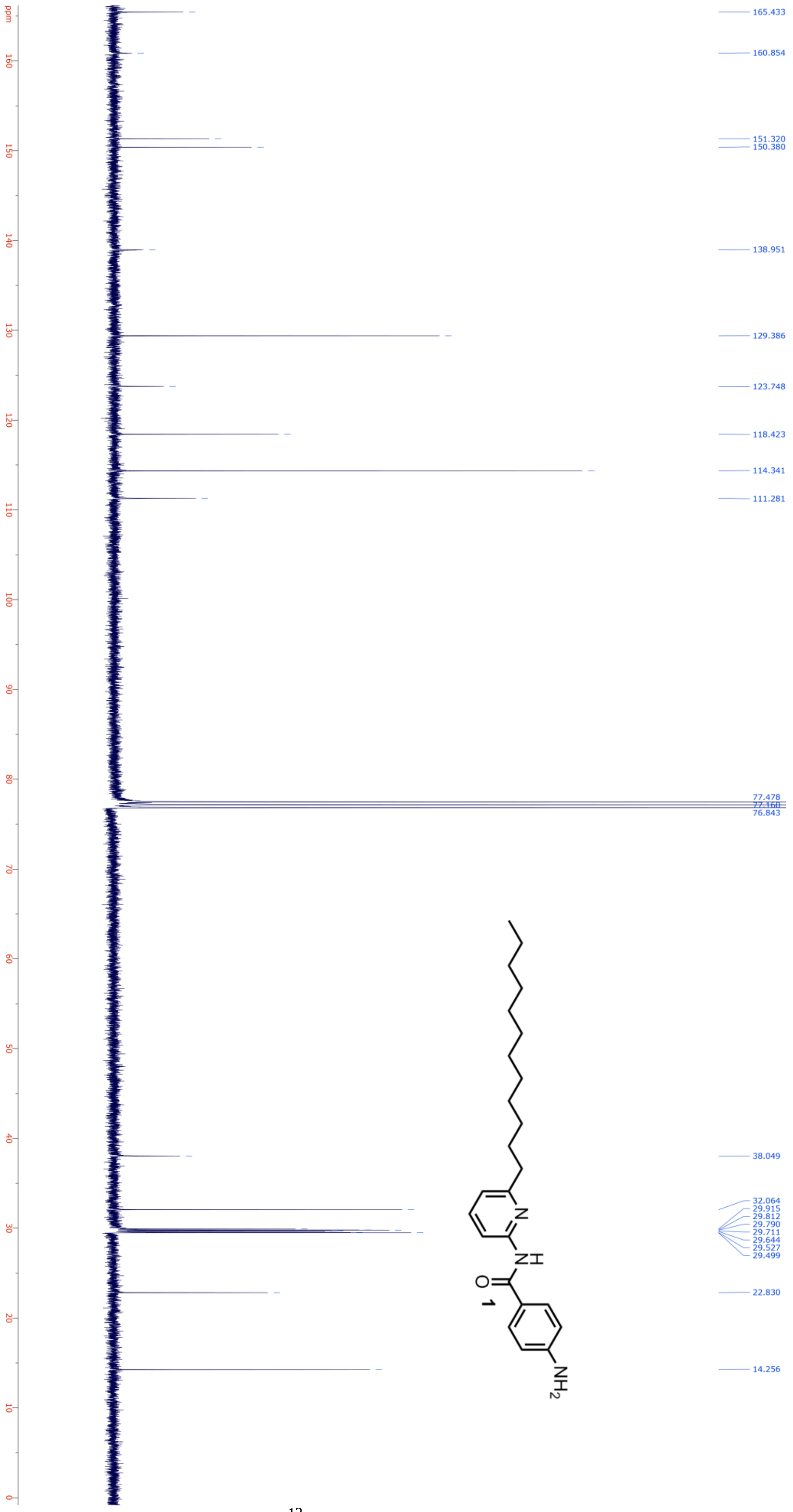

Supplementary Figure 12 – ^{13}C NMR spectrum of **1**

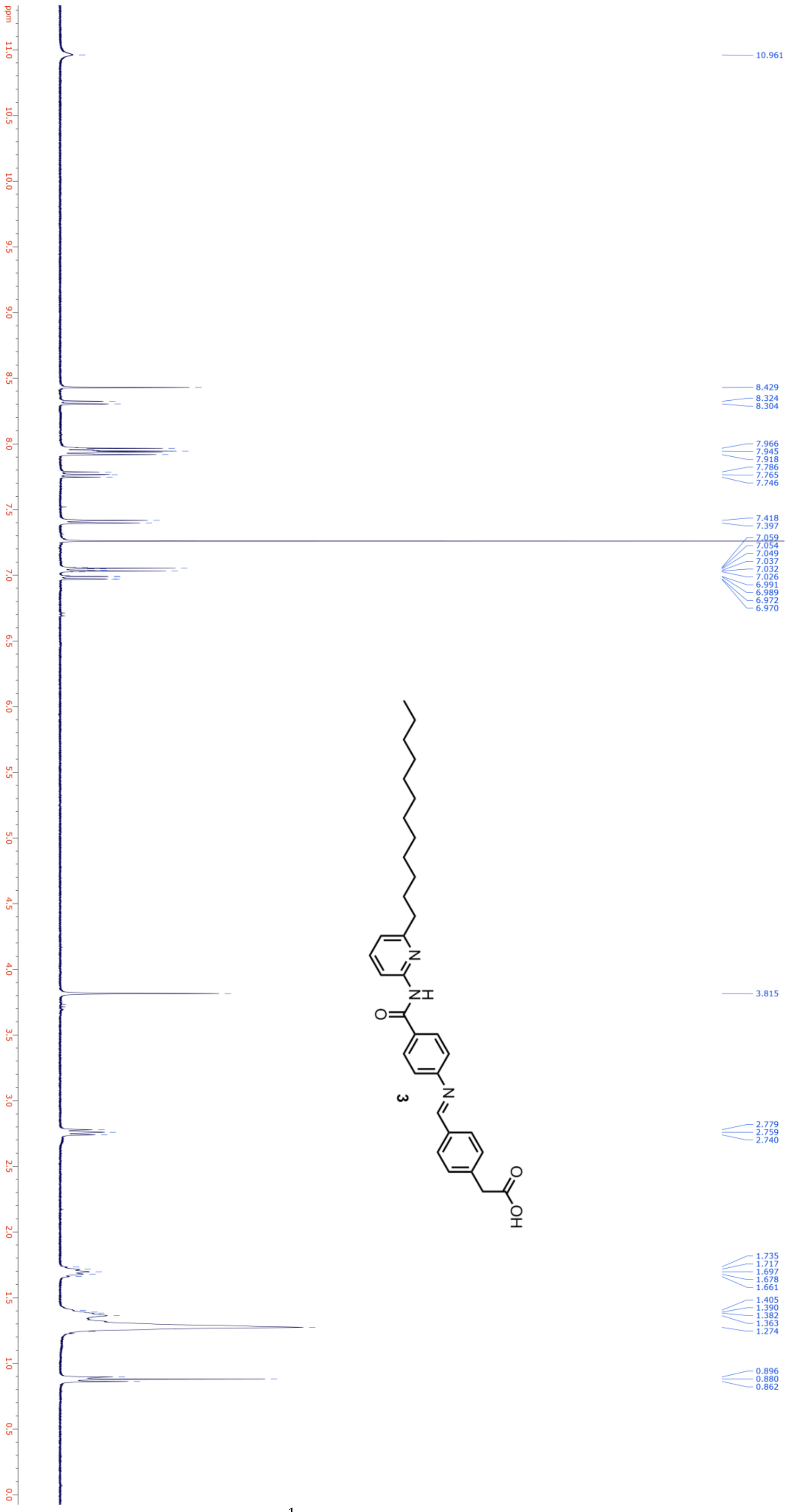

Supplementary Figure 13 – ^{1}H NMR spectrum of **3**

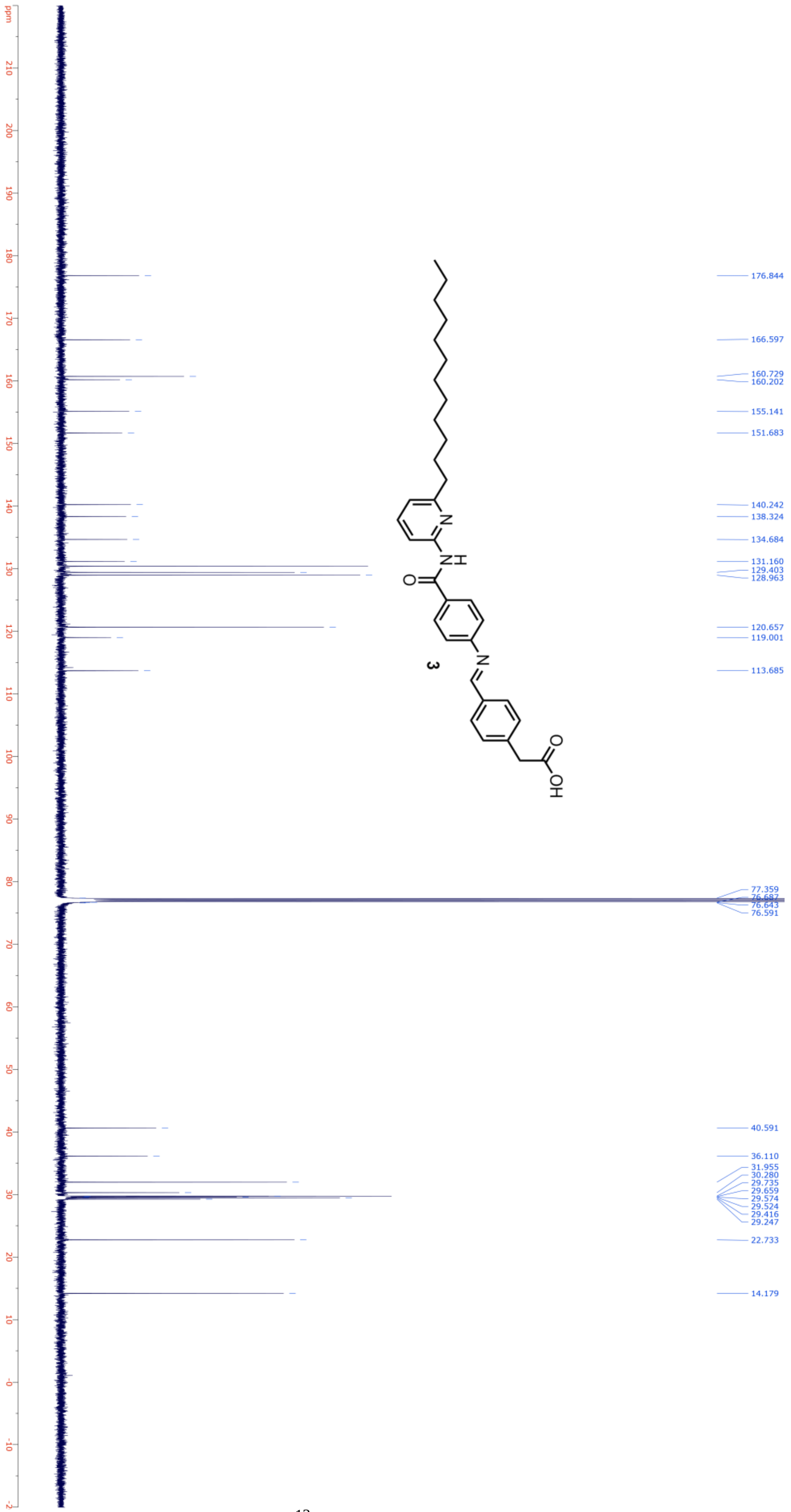

Supplementary Figure 14 – ^{13}C NMR spectrum of **3**

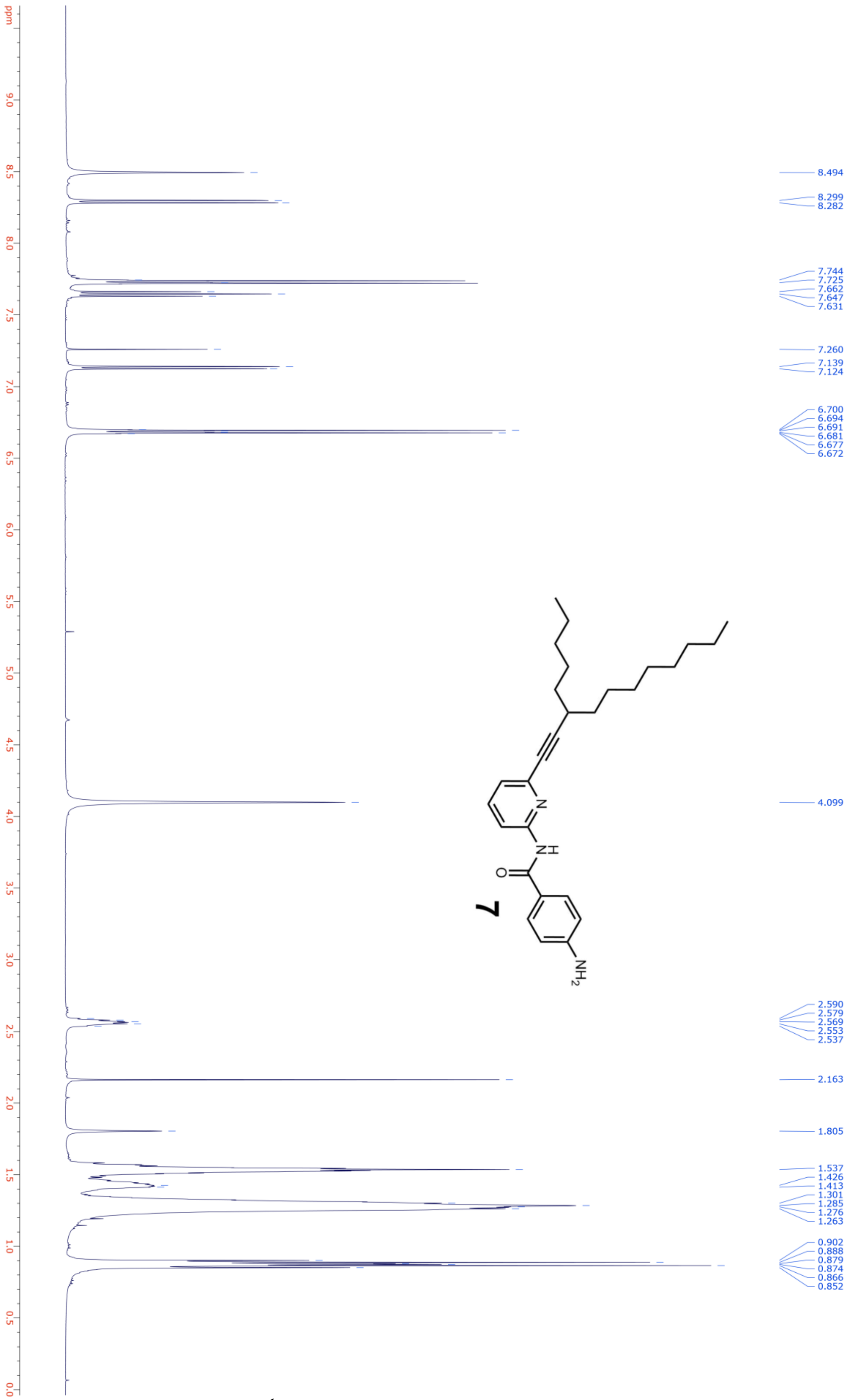

Supplementary Figure 15 – ^{1}H NMR Spectrum of **7**

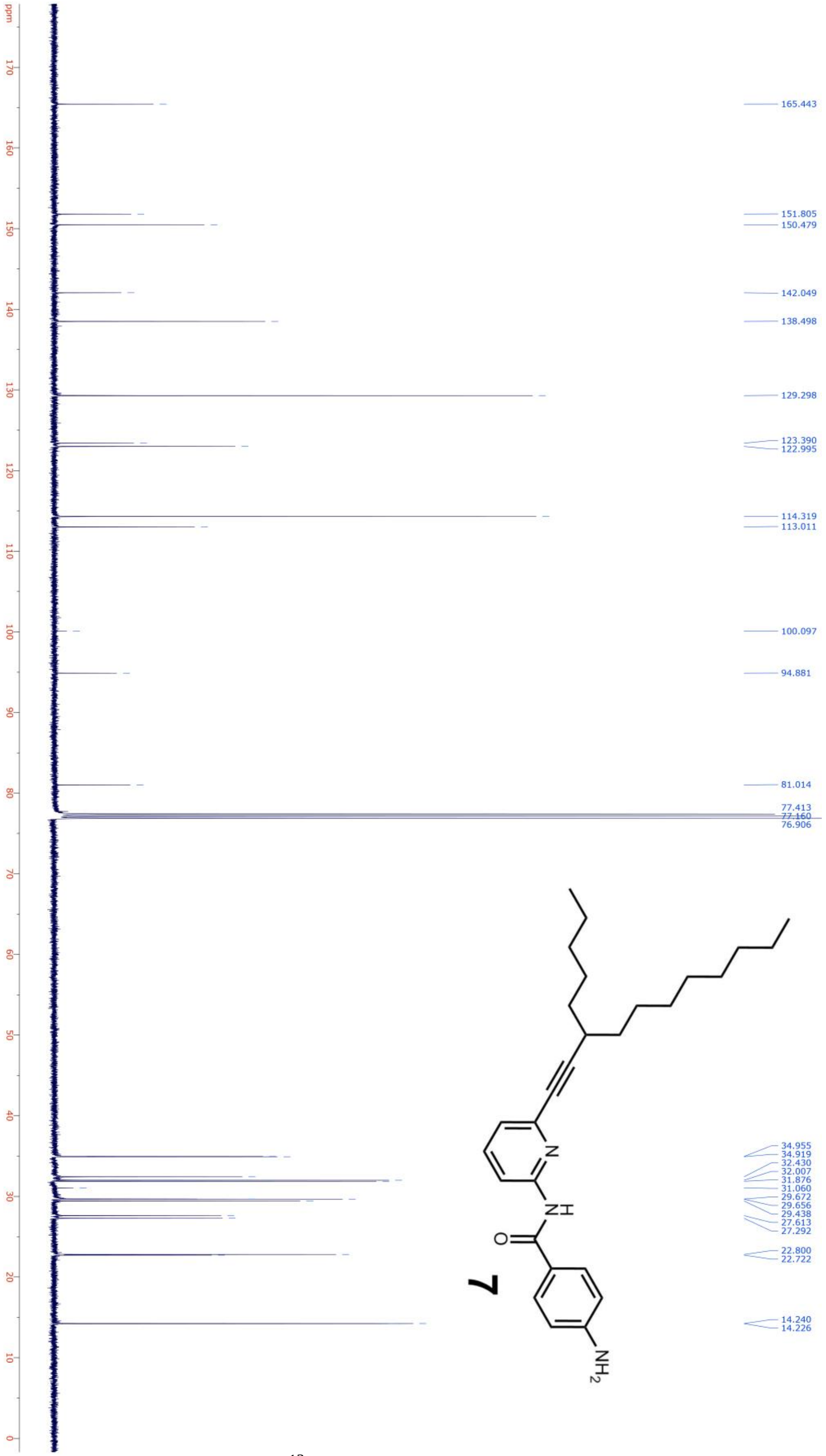

Supplementary Figure 16 - ^{13}C NMR Spectrum of **7**

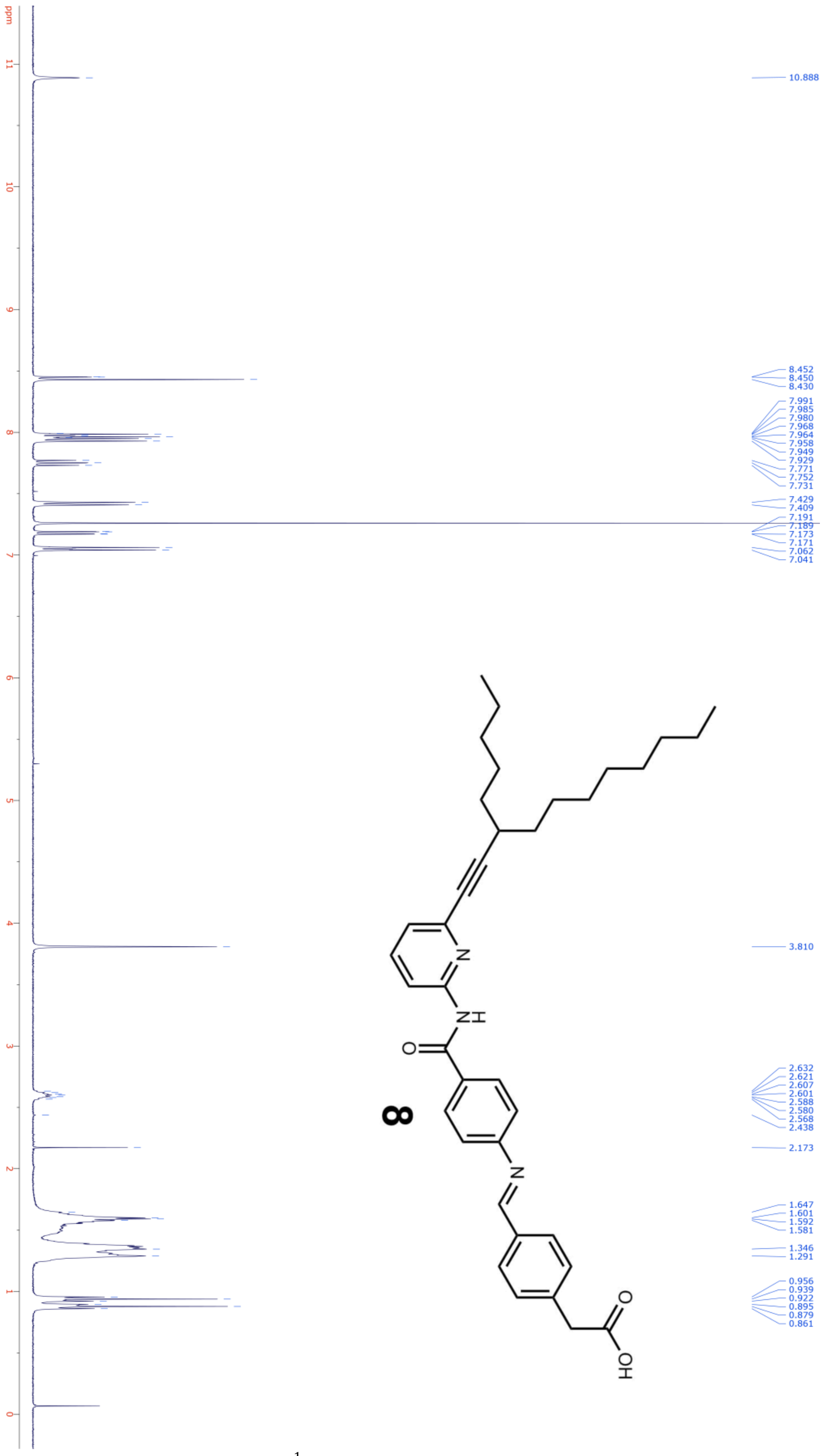

Supplementary Figure 17 - ^{1}H NMR Spectrum of **8**

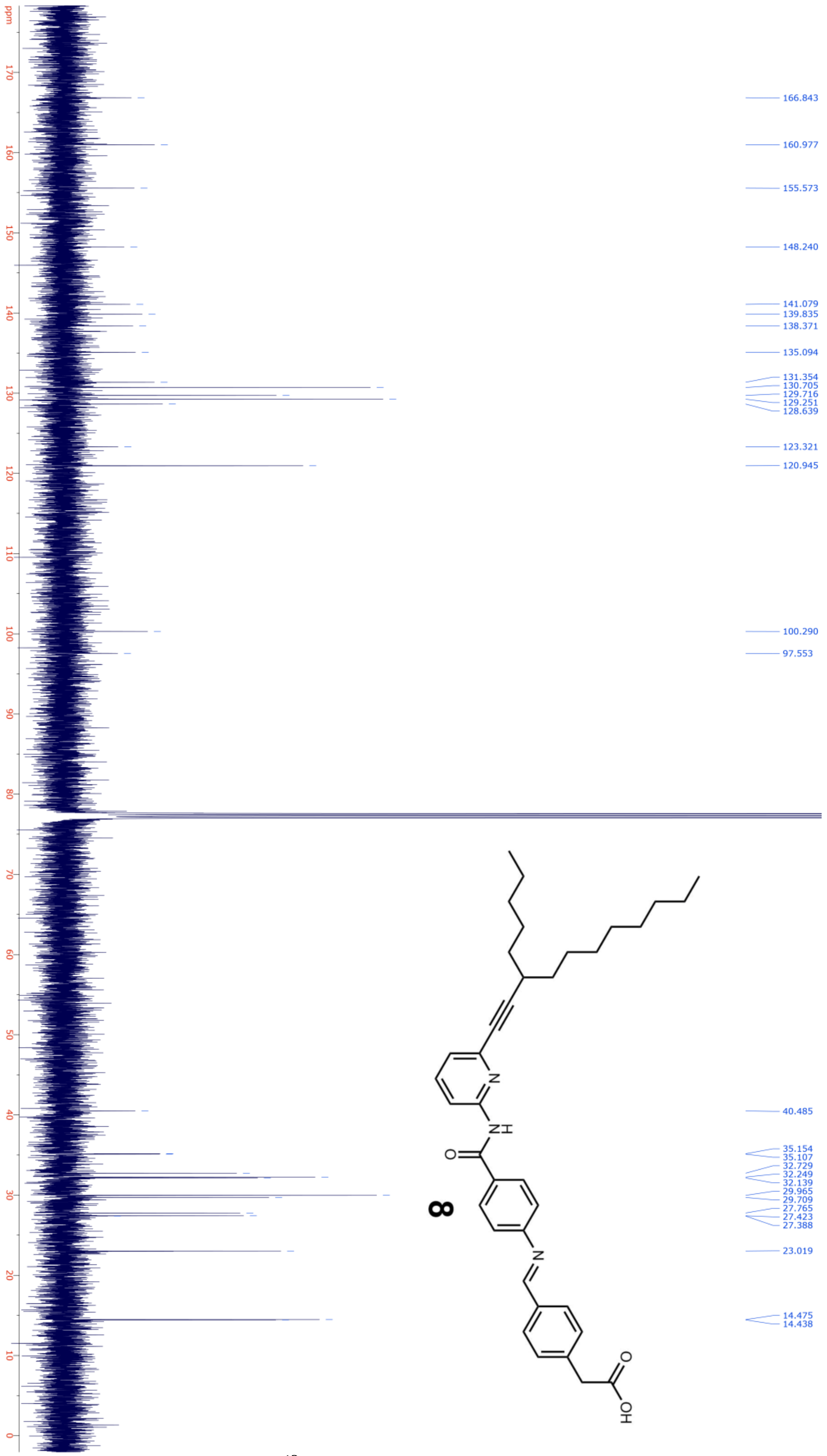

Supplementary Figure 18 - ^{13}C NMR Spectrum of **8**

Parameter	Unseeded	Seeded	Unmodified
k_1f ($M^{-1}s^{-1}$)	5.9×10^{-4}	1.3×10^{-3}	7.0×10^{-4}
k_6f ($M^{-1}s^{-1}$)	1.7×10^{-2}	1.6×10^{-2}	8.0×10^{-3}
k_6r ($M^{-1}s^{-1}$)	2.2×10^{-3}	1.3×10^{-3}	1.0×10^{-3}
K_{tt} (M^{-1})	80000	92000	40000
$K_{complex}$ (M^{-1})	34	25	45

Supplementary Table 1

Best fit values for rate and equilibrium constants in self-replication reactions.
(given to 2 *sf*)

Volume of water added (μl)	I (initial) after sonication (kcps)
**0**	62
**1.25**	1586
**2.5**	2777
**5**	37800

Supplementary Table 2

Increase in scattered intensity with increased water addition after the reaction between **1** and **2** had taken place.
